# Analyzing Disparity in Geographical Accessibility to Home Medical Care Using a Claims Database and Geographical Information System: Simulation Study

**DOI:** 10.2196/70040

**Published:** 2025-08-06

**Authors:** Yasuhiro Morii, Yasuhiro Nakanishi, Yuichi Nishioka, Yukio Tsugihashi, Tatsuya Noda, Tomoya Myojin, Tomoaki Imamura, Manabu Akahane

**Affiliations:** 1 Center for Outcomes Research and Economic Evaluation for Health, National Institute of Public Health, Wako, Japan; 2 Department of Health and Welfare Services, National Institute of Public Health, 2-3-6 Minami, Wako, 351-0197, Japan, +81 484586347, +81 484687985; 3 Department of Public Health, Health Management and Policy, Nara Medical University, Kashihara, Japan; 4 Medical home care center, Tenri Hospital Shirakawa Branch, Tenri, Japan; 5 Department of Community Health and Preventive Medicine, Hamamatsu University School of Medicine, Hamamatsu, Japan

**Keywords:** home medical care, geographical accessibility, geographical information system, medical claims database, simulation

## Abstract

**Background:**

The demand for home medical care services has increased in aging societies. Therefore, allocating health care resources optimally to meet the needs of each community is essential. Geographical accessibility is an important factor affecting access to home medical care services; however, little research has been conducted on regional disparities in geographical accessibility.

**Objective:**

This study aims to analyze the regional disparities in geographical accessibility to home medical care services using the Kokuho database (KDB), a comprehensive medical claims database for a prefecture in Japan.

**Methods:**

This study included 39 municipalities in Nara Prefecture, Japan. Using a geographical information system, accessibility to home medical care services, that is, travel distance and time from hospitals and clinics to hypothetical patients, was analyzed in two scenarios: (1) an ideal scenario, where we assumed that all hospitals or clinics in Nara Prefecture provided those services and (2) an actual scenario, where hospitals or clinics in Nara Prefecture that actually provided home medical care services, identified from KDB data analysis, were used in the analysis. Hypothetical patients were randomly distributed on the geographical information system in accordance with the usage rates of home medical care services and with the distributions of the population aged ≥75 years. The usage rate by municipalities was aggregated from the analysis of KDB data of Nara Prefecture in FY2019.

**Results:**

The median travel distance was longer than 16 km, the reference limit value specified in the Japanese fee table, and the median travel time exceeded 30 min in certain rural municipalities in the southern part of Nara Prefecture, in the actual scenario, whereas the travel distance and time were improved in the ideal scenario. The differences in travel time between the ideal and actual scenarios were the largest in the depopulated municipalities in the southern part, such as Totsukawa (32.6 vs 5.8 min), Kawakami (30.1 vs 11.8 min), Kurotaki (21.3 vs 5.2 min), and Kamikitayama (20.7 vs 3.5 min). The usage rates were also lower in rural municipalities in the southern part.

**Conclusions:**

The results revealed that geographical accessibility was lower in depopulated municipalities in the southern part, and the disparity could be partly solved in the ideal scenario, especially in that area, highlighting the necessity of increasing supply in the southern areas. KDB is a comprehensive database that includes medical claims information for home medical care patients and details of the provision of medical institutions, enabling geographical analysis that reflects actual health care usage.

## Introduction

Most limited-income countries have aging societies, including Japan, which is the most aging country in the world with an aging rate (rate of population aged ≥65 y) of 28.1% in 2018, followed by Italy (23.3%), Portugal (21.9%), and Germany (21.7%) [1]. Medical demand is estimated to increase with demographic changes [2]. The Japanese government has been promoting a structure in which home medical care and long-term care services are provided comprehensively to older adults, ensuring they live their lives in the areas they are used to in an aging society [3]. Therefore, increased promotion of the use of home medical care services is of great societal importance.

The demand for home medical care services has also increased in recent years, with a peak at around 2040 [3]. Each prefecture in Japan is responsible for creating a Regional Medical Plan to ensure that health care services are effectively provided to meet the needs of the local population [4]. The Regional Medical Plan includes a plan to provide home medical care services, and each prefecture must specify the problems faced and measures to resolve them. Prefectures must consider optimal allocations and make the most of their limited resources to meet the needs.

Geographical accessibility is among the factors that could affect the provision of home medical services. The relationship between geographical factors and the usage of medical services has been previously studied [5-7]. For example, Schwarz et al [5] analyzed the relationship between the distance from hospitals to patients and the psychiatric treatments that patients received. Herein, people living in peripheral communities were mainly treated in inpatient settings. In contrast, those living in residential areas were more likely to receive intensive psychiatric home treatment. Similar to their study findings, geographical accessibility is important for home medical care services since health care providers need to travel to patients’ homes.

Kuwayama et al [8] conducted a literature review of studies on home care nursing in Japan using a geographical information system (GIS) and discovered a few related studies [9,10]. In those studies, Naruse et al [7] performed a multilevel logistic analysis of the association between the use of home care nursing and the proportion of older adults reachable by facilities providing home-visit nursing services (calculated by buffer analysis using GIS). They found that people living in less reachable municipalities were less likely to use these services. However, few studies have been conducted on the geographical accessibility of home-based medical care services. Moreover, these previous studies were limited to visualizing the current situation.

Extensive databases containing medical claims and national health insurance information, such as the National Database of Health Insurance Claims and Specific Health Checkups of Japan (NDB) and National Health Insurance Database (Kokuho database: KDB), have recently become essential tools for medical and health care policy research. These real-world data sources provide valuable insights into health care usage, treatment patterns, and outcomes. The main advantages of using such databases include the ability to study targeted populations and understand the actual clinical practice comprehensively. Using such databases, a geographical analysis that reflects the actual health care usage and trends will be possible.

Several studies have conducted geographical analysis using a large real-world database. Cheng et al [11] analyzed the prescribing patterns of cardiovascular drugs across townships in Taiwan using a national claim database covering 97.5% of the total population. Kwak et al [12] analyzed the relationship between the incidence of obstetric complications for pregnant women and access time to delivery units in South Korea, using the national claim database.

However, there are few previous studies analyzing geographical accessibility to home medical care services using an extensive database.

Therefore, this study aimed to develop a methodology for analyzing regional disparities in geographical accessibility to home medical care services using the KDB database to help consider achieving the provision of more efficient and equitable service. Given the global trend of aging, the findings of this study in Japan may contribute to improving the efficiency and equity of service delivery and policymaking of home medical care services in other countries facing similar demographic challenges.

## Methods

### Subjects and Scheme

**Figure 1.**
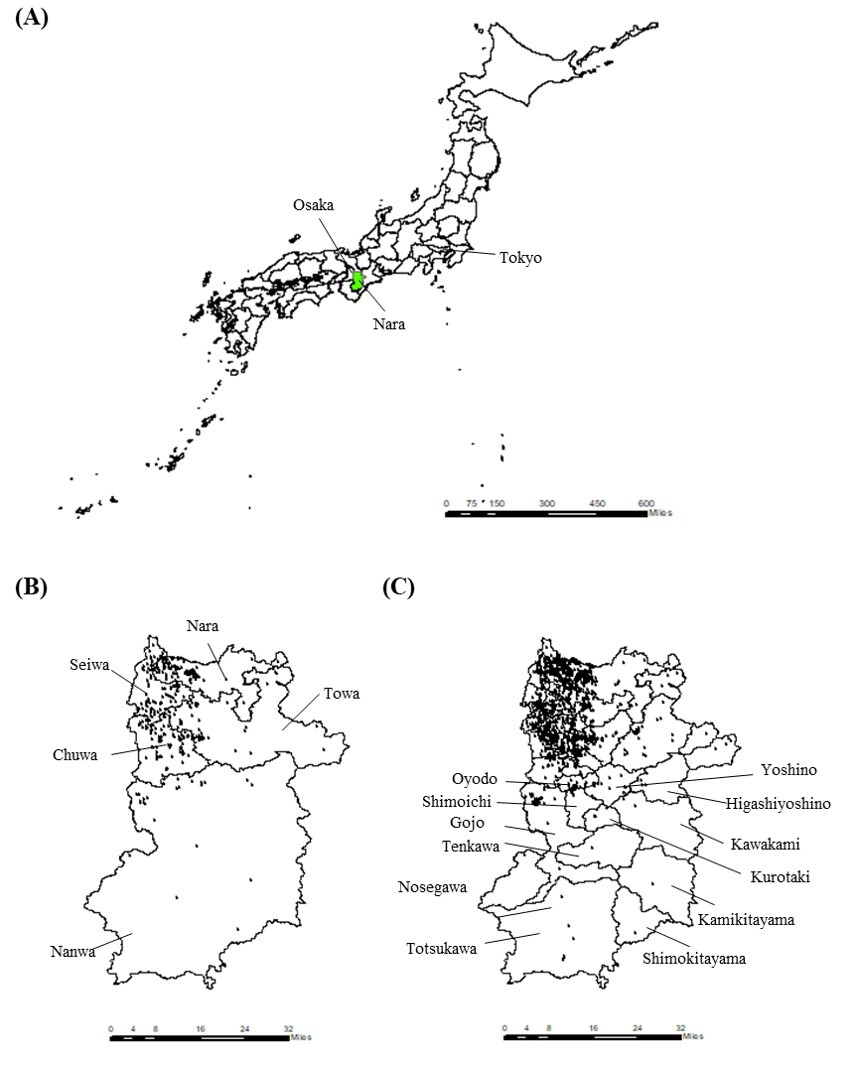
The geographical locations of Nara Prefecture in Japan, secondary medical areas in Nara, and municipalities in Nara show the geographical location of (A) Nara Prefecture in Japan and also illustrate the distributions of hospitals or clinics (B) in the actual scenario and (C) in the ideal scenario.

This study was conducted in the 39 municipalities in Nara Prefecture, Japan, as a model region. Nara Prefecture is located in the center of Japan’s main island of Honshu (Figure 1A) and has both urban and rural (mountainous) areas. Nara Prefecture has a population of 1,286,651 (approximately 1/100th of the national total), a land area of 3691 km^2^ (approximately 1/100th of the national total) [13,14], and 32% (0.4/1.25) of adults aged ≥65 years (compared to 36.4/125.52, 29% nationally; Table 1). The characteristics of municipalities, such as population and aging rate, are shown in Table 1. Figures 1B and C illustrate the geographical locations of the 5 secondary medical areas and 39 municipalities. Nanwa secondary area, in the southern part, is the most rural area with mountains. Since there seems to be a geographical difference within Nara Prefecture, it would be suitable as a model region for geographical analysis. Secondary medical areas provide general inpatient medical services, whereas municipalities or smaller regional units provide more basic medical services.

**Table 1.** Characteristics of each municipality and the result of usage rate.

Municipality	Population [14]	Aging rate (%) [14]	Land area (km^2^) [13]	Population density (/km^2^)	Number of claims	Usage rate (%)
Nara	347,947	32.5	276.9	1256.40	34,335	6.8
Kashihara	118,344	30	39.6	2991.50	5370	3.9
Ikoma	114,508	30	53.2	2154.40	7755	5.8
Yamatokoriyama	80,760	34.4	42.7	1891.80	5135	4.6
Kashiba	77,212	24.8	24.3	3182.70	3931	5.4
Tenri	60,717	27.9	86.4	702.6	4160	5.8
Yamatotakada	59,797	32.9	16.5	3628.50	3838	4.4
Sakurai	52,975	33.1	98.9	535.6	3812	5.4
Katsuragi	37,199	28.1	33.7	1103.20	1424	3.4
Koryo	33,910	27.3	16.3	2080.40	1154	3.6
Tawaramoto	30,840	32.6	21.1	1462.30	1497	4.9
Ikaruga	27,408	30.7	14.3	1920.70	2108	6.0
Uda	25,732	44.5	247.5	104	2103	4.7
Gojo	25,487	42.4	292	87.3	1769	4.2
Oji	23,667	28.9	7	3376.20	1518	5.4
Sango	22,688	33	8.8	2581.10	2018	7.2
Gose	22,259	43.9	60.6	367.4	1096	2.6
Kanmaki	20,815	37	6.1	3390.10	1586	5.3
Heguri	17,545	39.9	23.9	734.1	2484	7.9
Kawai	16,314	40.2	8.2	1982.30	1523	5.3
Oyodo	15,530	37.5	38.1	407.6	381	2.2
Kawanishi	7716	36	5.9	1301.20	457	3.6
Ando	6930	37.2	4.3	1607.90	439	4.9
Takatori	6235	43.6	25.8	241.8	274	2.5
Miyake	6097	37.2	4.1	1501.70	457	4.7
Yoshino	5457	54	95.7	57.1	407	2.7
Asuka	4767	43.2	24.1	197.8	238	3.1
Shimoichi	4356	49.7	62	70.3	154	2.0
Yamazoe	2909	51.6	66.5	43.7	289	4.4
Totsukawa	2695	43.4	672.4	4	254	3.6
Higashiyoshino	1337	60.5	131.7	10.2	224	5.1
Mitsue	1298	61.2	79.6	16.3	50	1.6
Soni	1175	53.5	47.8	24.6	140	5.0
Tenkawa	1048	53.3	175.7	6	60	1.5
Kawakami	1039	56.1	269.3	3.9	112	2.7
Shimokitayama	690	47.9	133.4	5.2	39	2.1
Kurotaki	536	54	47.7	11.2	24	0.9
Kamikitayama	384	49.6	274.2	1.4	37	2.6
Nosegawa	328	51.6	154.9	2.1	62	10.5
Total	1,286,651	32.7	3690.9	348.6	92,714	5.3

For each municipality, geographical accessibility to home medical care services was analyzed using GIS (ArcGIS Desktop version 10.8.1; ESRI Japan Corporation) in the following two scenarios: (1) actual scenario: geographical accessibility to patients from hospitals and clinics in Nara Prefecture that actually provided home medical care services, and (2) ideal scenario: geographical accessibility to patients from all hospitals or clinics in Nara Prefecture was used. The latter simulates geographical accessibility in an ideal scenario in which the number of hospitals or clinics that provide home medical care services would increase. These scenarios were different only in terms of the number of hospitals and clinics used in the analysis, as described hereafter. Hypothetical patients were generated on a GIS by using the methodology described in a subsequent section “Geographical Analysis.”

### Data

For the actual scenario, hospitals and clinics that provided home medical care services were defined using data from the KDB database of Nara Prefecture (Nara KDB) in FY2019. This is a prefecture-wide database of medical and long-term care insurance claims. To be defined as hospitals and clinics that actually provided home medical care services, hospitals and clinics were required to have at least one claim with home medical care service fees (medical service code: 114001110, 114030310, 114042110, 114042210, 114042810, 114046310, 114027710, and 114027810 in the national fee schedule) for patients aged ≥75 years in Nara KDB in FY2019. Consequently, 346 hospitals and clinics were identified in the actual scenario.

The ideal scenario used all the hospitals and clinics in Nara prefecture, that is, those providing or not providing home medical care services. The geographical data on the 1308 hospitals and clinics were obtained from the Geospatial Information Authority of Japan [15]. Dental clinics were excluded from the study. The distribution of hospitals and clinics in the 2 scenarios is shown in Figure 1.

Population data at each 500 m^2^ mesh (ESRI Japan Corporation) were used to distribute the locations of the hypothetical patients.

### Geographical Analysis

First, hypothetical patients were generated on GIS. The number of patients in each municipality was that of patients aged ≥75 years who used home medical care services in each municipality, identified from the Nara KDB data analysis. The usage of home medical care was defined in the same manner as identifying the hospitals and clinics in the actual scenario. That number of patients was randomly allocated to 500 m^2^ meshes on GIS, reflecting the distribution of the population aged ≥75 years based on the national census 2020. In other words, the hypothetical patients were allocated to each mesh in a manner that the number of patients in each mesh is proportional to the mesh population aged ≥75 years using R version 4.1.2 (The R Foundation) [16,17]. This methodology enables the simulation of accessibility, reflecting the actual geographical distribution of potential patients. Thereafter, based on the patient allocations, hypothetical patient points were created on randomly determined points within the allocated meshes on ArcGIS using the “Create Random Points” function.

Subsequently, travel time from the patients to the hospitals or clinics was analyzed using the “Find Closest Facilities” function of ArcGIS for the 2 scenarios, respectively. We assumed that the travel to the patients was from the closest hospital or clinic. However, the closest hospitals or clinics can be located outside the municipality where the patients live.

The median and 25‐75 percentiles of travel distance and time were aggregated for the patients in each municipality. Moreover, the rates of patients for whom travel distances were over 16 km were calculated since, in the Japanese universal fee table, the travel distance to provide home medical care services needs to be 16 km or less, unless an exceptional reason is present [18].

**Figure 2.**
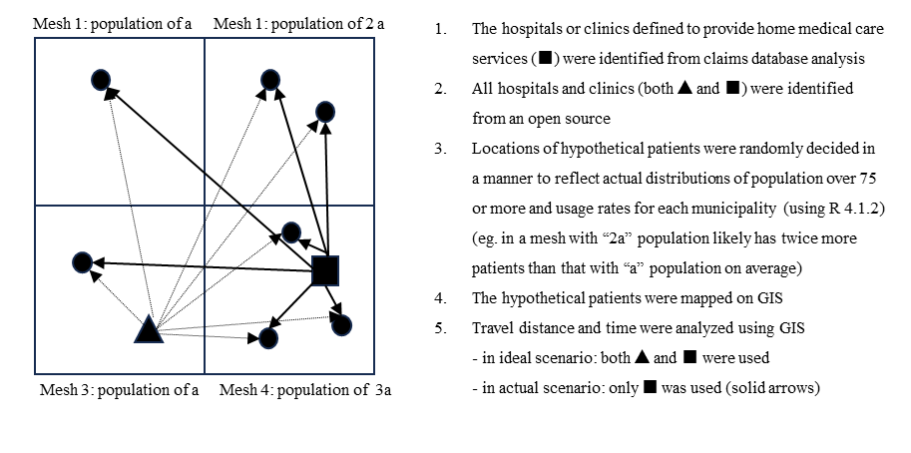
Scheme of the geographical analysis illustrating the scheme of the geographical analysis. GIS: geographical information system.

The usage rate was calculated by dividing the number of patients aged ≥75 years using home medical care services by the total population aged ≥75 years. The entire process of geographical analysis was repeated 10 times to consider the uncertainty of patient locations in each trial. The scheme of geographical analysis is visualized in Figure 2.

### Ethical Considerations

This study was approved by the ethics committee of the National Institute of Public Health, Japan (NIPH-IBRA number 12324‐2). The requirement for informed consent was waived because all data were anonymized.

## Results

**Figure 3.**
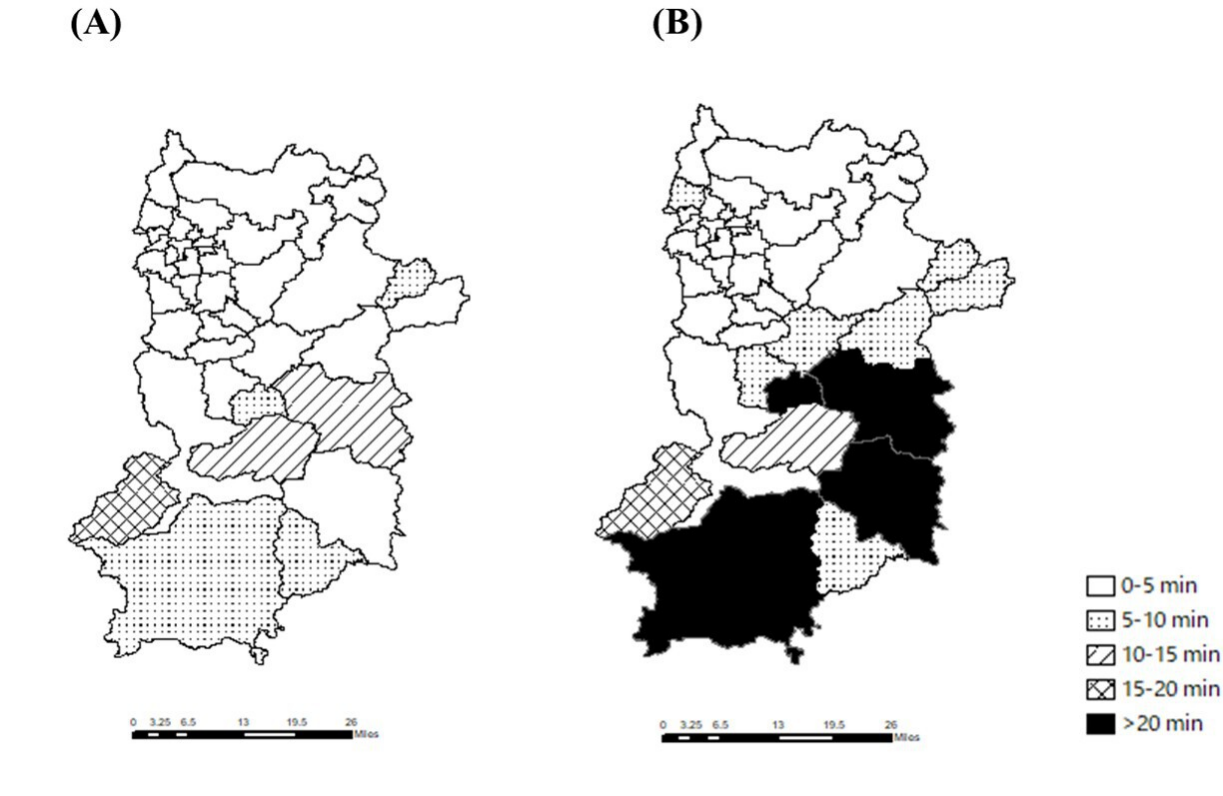
The median travel time in the two scenarios. This figure illustrates the median travel time in (A) the ideal scenario and (B) the actual scenario.

**Figure 4.**
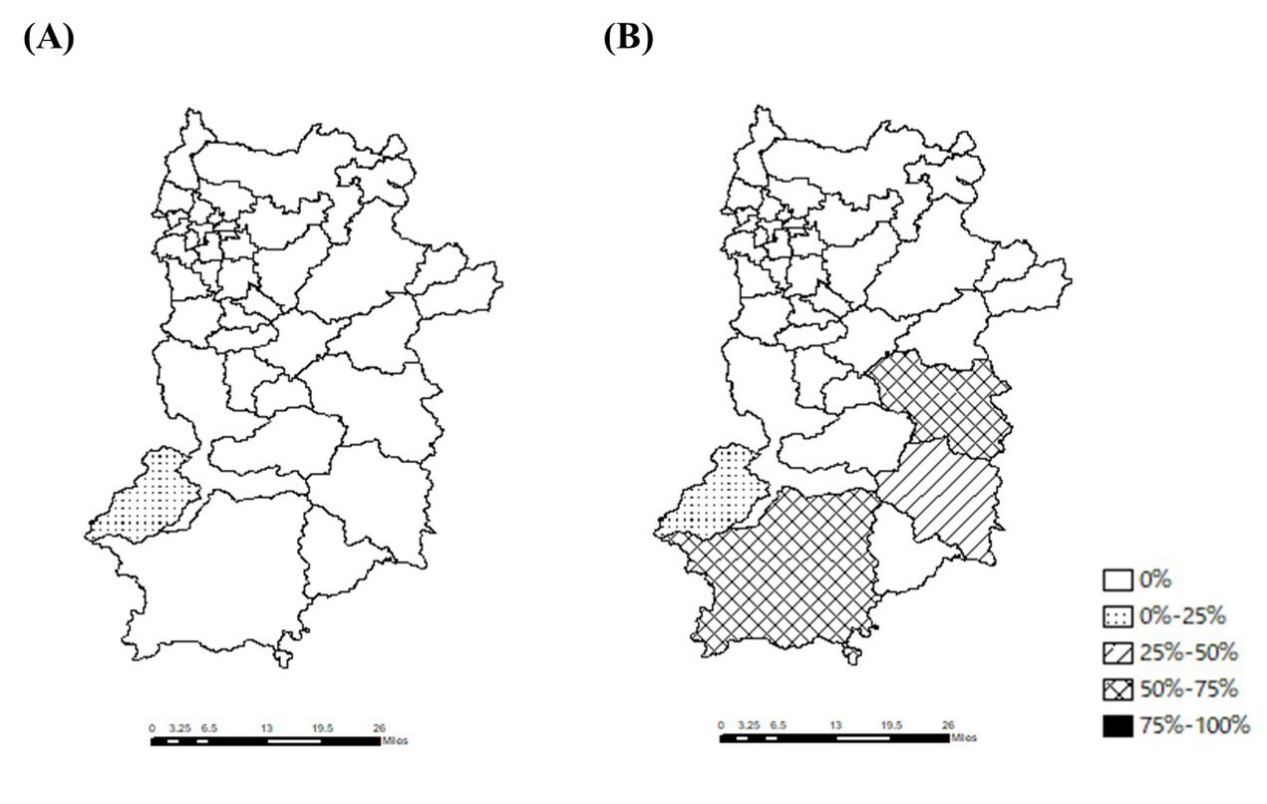
The rates of patients for whom the travel time was longer than 30 minutes. This figure illustrates the rates of patients for whom the travel time was longer than 30 minutes in (A) the ideal scenario and (B) the actual scenario.

**Figure 5.**
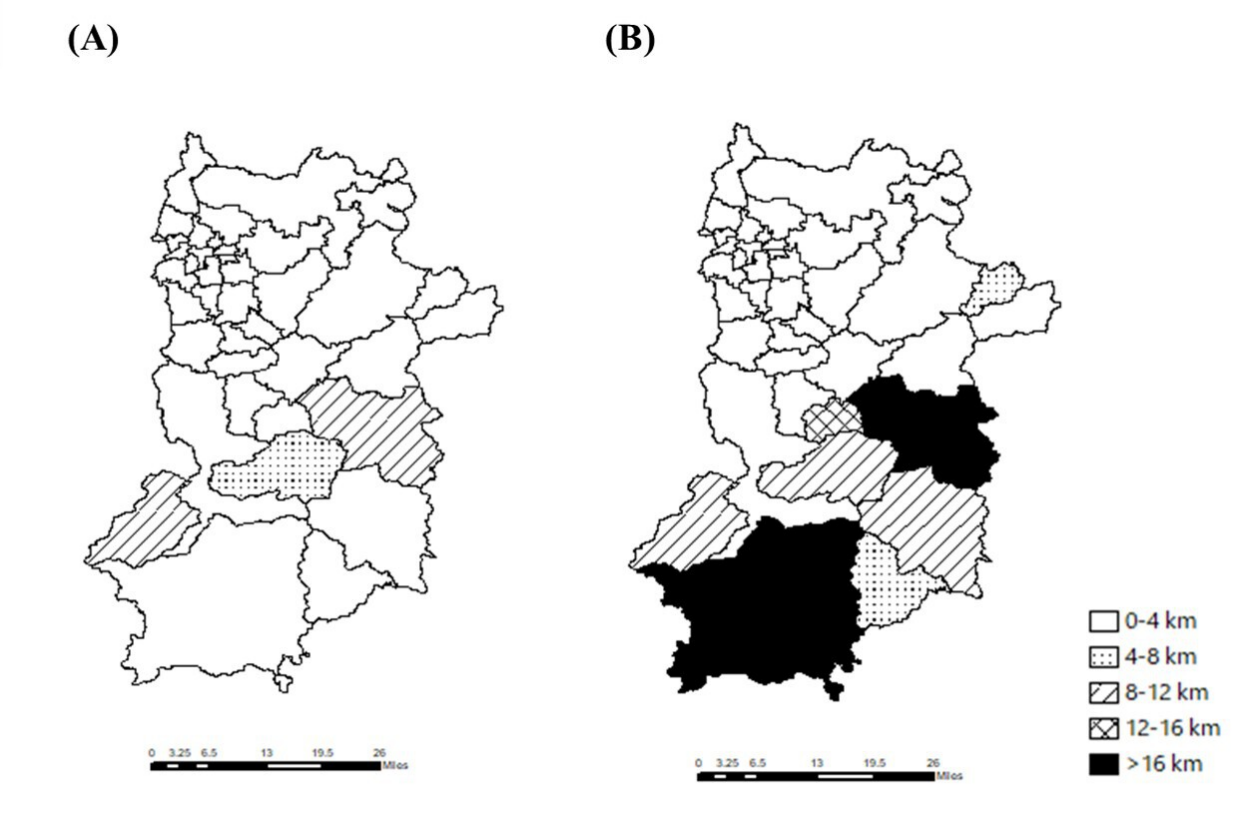
The median travel distance for each municipality in the two scenarios illustrates the median travel distance in (A) the ideal scenario and (B) the actual scenario.

**Figure 6.**
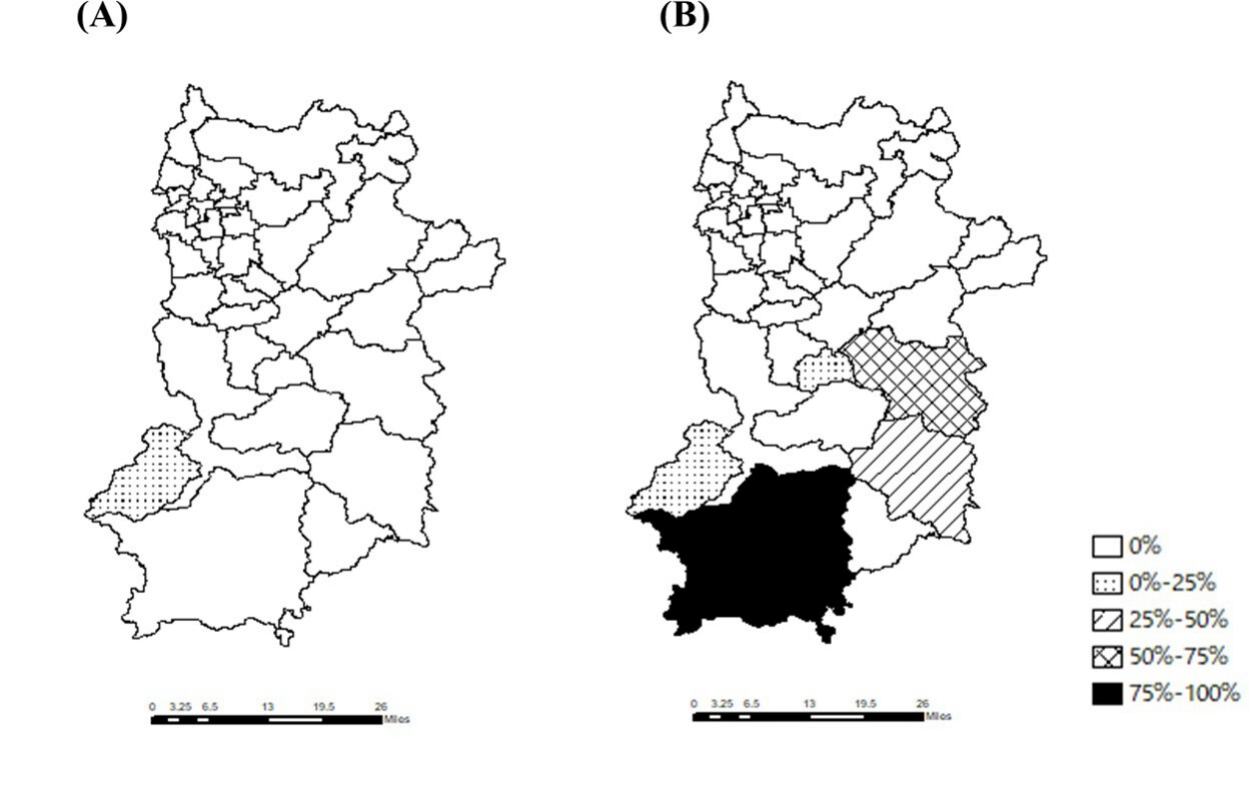
The rates of patients for whom the travel distance was longer than 16 km. This illustrates the rates of patients for whom the travel distance was longer than 16 km in (A) the ideal scenario and (B) the actual scenario.

The number of claims and usage rates are presented in Table 1. The distributions of hospitals or clinics used in each scenario were visualized in Figure 1. The travel time and distance results are shown in Figures 3-6 and MultimediaAppendices 1  2. In the actual scenario, the median travel time for all the patients in Nara Prefecture was 3.0 (IQR 2.0‐4.3) minutes, and the travel distance was 0.7 (IQR 0.5‐1.2) km. In the ideal scenario, the median travel time for all patients was 2.0 (IQR 1.2‐2.9) minutes, and the travel distance was 0.5 (IQR 0.3‐0.9) km.

The median travel times for each municipality in the 2 scenarios are shown in Figures3  4. In the ideal scenario, the median travel time was less than 15 minutes, except for Nosegawa (18.1, IQR 2.2‐25.5 min), while in the actual scenario, the median travel time was longer in the municipalities in the Nanwa area, the southern part (in Totsukawa (32.6, IQR 21.2‐40.5 min), Kawakami (30.1, IQR 16.5‐32.4 min), Kurotaki (21.3, IQR 20.2‐24.1 min), and Kamikitayama (20.7, IQR 19.7‐26.1 min; Figure 3).

When comparing the 2 scenarios, the differences in travel time between the scenarios were the largest in Totsukawa (26.8 min), Kawakami (18.3 min), Kamikitayama (17.2 min), and Kurotaki (16.1 min). All of these 4 municipalities are located in a depopulated secondary medical area of Nanwa.

Figure 4 shows the number of patients for whom the travel time was longer than 30 minutes. In the actual scenario, the rates were highest in Totsukawa (58.0%), Kawakami (50.0%), Kamikitayama (30.0%), and Nosegawa (25.0%).

The median travel distances for each municipality in the 2 scenarios are shown in Figure 5. In the ideal scenario, the median travel distances were shorter than 12 km for all municipalities. However, in the actual scenario, the median travel distances were longer than 16 km in Totsukawa (28.4, IQR 16.7‐32.3 km) and Kawakami (17.2, IQR 10.3‐19.3 km). Kurotaki, Nosegawa, and Kamikitayama showed similar findings (13.9, IQR 12.6‐14.4; 11.5, IQR 1.3‐16.1; and 10.8, IQR 10.3‐16.4 km, respectively). The differences in travel distance between the scenarios were the largest in Totuskawa (24.5 km), Kurotaki (11.8 km), Kamikitayama (9.3 km), and Kawakami (8.6 km).

Figure 6 shows the number of patients whose travel distance was >16 km. In the actual scenario, the rates were 77.6% for Totsukawa, 56.2% for Kawakami, 30.0% for Kamikitayama, 25% for Nosegawa, 20% for Kurotaki, and 0% for the other municipalities. In the ideal scenario, the rate was zero, except in Nosegawa (25.4%).

The usage rates by municipalities are shown in Table 1. This table shows the usage rates of home medical care services in each municipality. As can be seen, the usage rates tended to be lower in the municipalities in the Nanwa area (colored black), except for Nosegawa, which had the highest rate.

## Discussion

### Principal Findings

In this study, an accessibility analysis of home medical care services in Nara Prefecture was conducted as a model region, using the KDB data to investigate the equitable distribution of home medical care services. Using the KDB, we accurately identified medical institutions that provide home medical care, estimated the usage rates of home medical care services for each municipality, and demonstrated the feasibility of analyzing geographical accessibility to home medical care services that reflect actual health care usage and clinical trends.

The findings of this study revealed that in certain municipalities within the Nanwa area, a rural secondary medical area with a declining population, the travel distance to a health care patient’s home exceeded 16 km, and the travel time exceeded 30 minutes in some cases. These results highlight disparities among municipalities in terms of geographic accessibility to home medical care services. Its supply in the Nanwa area needs to be increased from the perspective of geographic accessibility.

In addition, the usage rates of these services were lower in rural municipalities in the Nanwa area. To promote the use of home medical care services, improving accessibility in these municipalities is necessary. Geographical accessibility—that is, the distance from service providers to patients—could affect service usage. Naruse et al [7] reported that travel distance can affect the usage of home nursing care services. Although geographical accessibility would also likely affect the usage of home medical care services, the extent of the effect has not been clear since this study did not quantify the effect by controlling other factors, such as socioeconomic status, care quality, and regional characteristics. Further studies with additional information beyond medical claim data are required to clarify this point.

In recent years, the government has promoted the use of home medical care to meet the increasing medical demands within each area, enabling local people to live their lives in areas they have always lived in [3]. Our results showed that a disparity occurred in geographical accessibility to home medical care based on the current practice in the actual scenario; however, in the ideal scenario, no municipality was observed in which the travel distance to any patient was longer than 16 km, except for Nosegawa. Geographical accessibility is much better in this scenario. For example, the average travel time was less than 15 minutes, except for Nosegawa. These results indicate that the disparity in terms of geographical accessibility would have been mostly resolved if all hospitals and clinics had provided these services. Furthermore, our results indicated that the potential benefit would be the largest in the Nanwa area, the most depopulated area. Therefore, if the number of hospitals or clinics providing these services is increased by a policy (eg, through dispatching health care professionals or funding), this will be effective in ensuring sufficient geographical accessibility for patients in the Nanwa area (specifically, Kawakami, Totsukawa, Kurotaki, and Kamikitayama). That is especially necessary in Kawakami and Kurotaki, since no hospitals or clinics were providing services, and the usage rates were low in these municipalities. However, it is neither feasible nor efficient for all hospitals and clinics to provide services because of limited resources. Further analysis using GIS could clarify how residents’ geographical accessibility will change when a specific hospital or clinic starts to provide home medical care services.

Notably, while the travel distance was longer in Nosegawa than in the other municipalities in both scenarios, the usage rate was the highest. The high usage rate could have been achieved by factors other than geographical accessibility. Further study, including factors other than geographical accessibility, is required to clarify the reason.

Our study is among the few that have analyzed geographical accessibility to home medical care services using GIS and KDB. The Nara KDB used in this study is an integrated database of medical and long-term care insurance claim information covering all residents aged 75 years and older, which matches the primary population of home medical care patients. This methodology enabled us to conduct a geographical analysis that reflected actual health care usage. Furthermore, our results are important when considering the optimal allocation of medical resources related to home medical care services and equal accessibility to these services. Japan is the most aging country in the world [1]. Home medical care services will be more important in other countries, and ensuring accessibility to these services will be of greater importance in the future. Geographical analyses using nationwide health care claims databases have been conducted in other countries for health care fields, such as drug prescription and pregnancy outcomes [11,12]. Likewise, our methodology can be applied to other countries for home medical care services, as those services will be more important as the aging of the population progresses.

### Limitations

The KDB is a comprehensive database that includes medical claims information for home medical care patients and details the provision of medical institutions, enabling a geographical analysis that reflects actual health care usage. However, this study had some limitations. First, it did not consider the capacity of each hospital or clinic because it focused on geographical accessibility. Further studies are required to consider its capacity to be more informative for optimal resource allocation. Second, a hospital or clinic was regarded as providing home medical care services if at least one claim regarding services from a hospital or clinic was identified. Some hospitals and clinics included in the actual scenario might not routinely provide home medical care services. Third, the disparity in geographical accessibility within each municipality is beyond the scope of this study. Such disparities may exist particularly in municipalities with lower geographical accessibility. Fourth, the KDB was used to analyze home medical care patients aged ≥75 years; therefore, home health care patients aged <75 years were not included. In the future, using the NDB or KDB and applying the developed methods, the analysis could be expanded to include pediatric home medical care patients and those under the age of 75 years. Finally, this study only used KDB data from FY 2019 to conduct geographical analysis while excluding the impact of the COVID-19 pandemic. The usage of home medical care has been increasing over the years, and the usage patterns could also change over the years. By applying the methodology established in this study, a nationwide analysis using the most recent data, free from the influence of the COVID-19 pandemic, can provide valuable insights into the future of home medical care.

### Conclusions

This study conducted a geographical accessibility analysis of home medical care services using Nara Prefecture as a model region with GIS and KDB. The results revealed that geographical accessibility was lower in depopulated municipalities in the southern part, such as Totsukawa, Kawakami, Kamikitayama, and Nosegawa, which have lower accessibility than urban areas. However, the results also showed that the disparity could be partly solved in the ideal scenario. The differences in travel time between the scenarios were especially larger in the depopulated areas in the southern part, such as Totsukawa (32.6 vs 5.8 min), Kawakami (30.1 vs 11.8 min), Kurotaki (21.3 vs 5.2 min), and Kamikitayama (20.7 vs 3.5 min). These results highlight the necessity of increasing supply in the southern areas.

## Supplementary material

10.2196/70040 Multimedia Appendix 1 Median and 25-75 percentiles of travel distance in the ideal and actual scenarios (km).

10.2196/70040 Multimedia Appendix 2 Median and 25-75 percentiles of travel time in the ideal and actual scenarios (min).

## Abbreviations

GIS: geographical information system
KDB: Kokuho database
NDB: National Database of Health Insurance Claims and Specific Health Checkups of Japan
