# Supplementary material for: Analyzing Disparity in Geographical Accessibility to Home Medical Care Using a Claims Database and Geographical Information System: Simulation Study
Source: JMIR Aging. 2025 Aug 6;8:e70040. doi: 10.2196/70040 (PMC12327904; doi:10.2196/70040)
Supplement: Multimedia Appendix 1 [file aging-v8-e70040-s001.docx]

Median and 25-75 percentiles of travel distance in the ideal and actual scenarios (km)

|  | ideal scenario | | | | actual scenario | | | |
| --- | --- | --- | --- | --- | --- | --- | --- | --- |
| city | 25% | 50% | 75% | over 16km | 25% | 50% | 75% | over 16km |
| Nara | 0.2 | 0.4 | 0.6 | 0.0% | 0.4 | 0.6 | 0.9 | 0.0% |
| Yamatotakada | 0.2 | 0.4 | 0.7 | 0.0% | 0.4 | 0.7 | 1.0 | 0.0% |
| Yamatokoriyama | 0.2 | 0.4 | 0.7 | 0.0% | 0.4 | 0.7 | 1.0 | 0.0% |
| Tenri | 0.3 | 0.6 | 1.1 | 0.0% | 0.5 | 0.8 | 1.3 | 0.0% |
| Kashihara | 0.2 | 0.4 | 0.6 | 0.0% | 0.4 | 0.6 | 1.0 | 0.0% |
| Sakurai | 0.3 | 0.5 | 0.9 | 0.0% | 0.4 | 0.8 | 1.4 | 0.0% |
| Gojo | 0.3 | 0.7 | 1.5 | 0.0% | 0.6 | 1.1 | 2.7 | 0.0% |
| Gose | 0.3 | 0.6 | 1.0 | 0.0% | 0.6 | 1.1 | 2.3 | 0.0% |
| Ikoma | 0.3 | 0.5 | 0.7 | 0.0% | 0.5 | 0.7 | 1.0 | 0.0% |
| Kashiba | 0.2 | 0.4 | 0.7 | 0.0% | 0.4 | 0.7 | 1.0 | 0.0% |
| Katsuragi | 0.3 | 0.4 | 0.7 | 0.0% | 0.5 | 0.7 | 1.0 | 0.0% |
| Uda | 0.4 | 0.8 | 2.0 | 0.0% | 0.9 | 1.7 | 3.9 | 0.0% |
| Yamazoe | 0.9 | 1.9 | 3.0 | 0.0% | 1.6 | 2.6 | 4.0 | 0.0% |
| Heguri | 0.5 | 0.8 | 1.1 | 0.0% | 1.1 | 1.5 | 2.0 | 0.0% |
| Sango | 0.4 | 0.6 | 0.9 | 0.0% | 0.6 | 0.9 | 1.2 | 0.0% |
| Igaruga | 0.3 | 0.4 | 0.6 | 0.0% | 0.5 | 0.7 | 0.9 | 0.0% |
| Ando | 0.7 | 0.8 | 1.0 | 0.0% | 1.2 | 1.4 | 1.5 | 0.0% |
| Kawanishi | 0.4 | 0.6 | 0.7 | 0.0% | 0.4 | 0.7 | 0.9 | 0.0% |
| Miyake | 0.4 | 0.7 | 0.9 | 0.0% | 0.4 | 0.7 | 1.0 | 0.0% |
| Tawaramoto | 0.3 | 0.6 | 0.8 | 0.0% | 0.5 | 0.9 | 1.3 | 0.0% |
| Soni | 1.1 | 3.3 | 4.9 | 0.0% | 2.4 | 5.2 | 6.9 | 0.0% |
| Mitsue | 0.8 | 1.8 | 3.3 | 0.0% | 2.7 | 3.9 | 5.3 | 0.0% |
| Takatori | 0.3 | 0.6 | 1.0 | 0.0% | 0.8 | 1.5 | 2.1 | 0.0% |
| Asuka | 0.4 | 0.7 | 1.2 | 0.0% | 1.0 | 1.4 | 2.0 | 0.0% |
| Kanmaki | 0.2 | 0.4 | 0.6 | 0.0% | 0.3 | 0.5 | 0.8 | 0.0% |
| Oji | 0.3 | 0.5 | 0.7 | 0.0% | 0.5 | 0.8 | 1.1 | 0.0% |
| Koryo | 0.3 | 0.5 | 0.7 | 0.0% | 0.7 | 1.0 | 1.3 | 0.0% |
| Kawai | 0.2 | 0.3 | 0.4 | 0.0% | 0.3 | 0.5 | 0.6 | 0.0% |
| Yoshino | 0.3 | 0.7 | 1.6 | 0.0% | 1.6 | 4.0 | 6.6 | 0.0% |
| Oyodo | 0.5 | 0.8 | 1.4 | 0.0% | 1.2 | 1.7 | 2.1 | 0.0% |
| Shimoichi | 0.8 | 1.5 | 3.9 | 0.0% | 1.4 | 2.2 | 4.7 | 0.0% |
| Kurotaki | 1.9 | 2.1 | 3.6 | 0.0% | 12.6 | 13.9 | 14.4 | 20.0% |
| Tenkawa | 2.3 | 7.6 | 8.3 | 0.0% | 3.0 | 8.2 | 8.9 | 0.0% |
| Nosegawa | 1.3 | 11.5 | 16.1 | 25.0% | 1.3 | 11.5 | 16.1 | 25.0% |
| Totsukawa | 1.1 | 3.9 | 6.7 | 3.0% | 16.7 | 28.4 | 32.3 | 78.0% |
| Shimokitayama | 1.2 | 3.7 | 6.1 | 0.0% | 3.0 | 5.6 | 6.7 | 0.0% |
| Kamikitayama | 0.5 | 1.5 | 6.5 | 0.0% | 10.3 | 10.8 | 16.4 | 30.0% |
| Kawakami | 3.9 | 8.6 | 10.4 | 1.0% | 10.3 | 17.2 | 19.3 | 56.0% |
| Higashiyoshino | 0.8 | 2.7 | 4.9 | 0.0% | 1.6 | 3.4 | 7.2 | 0.0% |
