# Supplementary material for: Analyzing Disparity in Geographical Accessibility to Home Medical Care Using a Claims Database and Geographical Information System: Simulation Study
Source: JMIR Aging. 2025 Aug 6;8:e70040. doi: 10.2196/70040 (PMC12327904; doi:10.2196/70040)
Supplement: Multimedia Appendix 2 [file aging-v8-e70040-s002.docx]

Median and 25-75 percentiles of travel time in the ideal and actual scenarios (minutes)

|  | ideal scenario | | | | actual scenario | | | |
| --- | --- | --- | --- | --- | --- | --- | --- | --- |
| city | 25% | 50% | 75% | over 30 minutes | 25% | 50% | 75% | over 30 minutes |
| Nara | 1.0 | 1.7 | 2.6 | 0.0% | 1.8 | 2.7 | 3.8 | 0.0% |
| Yamatotakada | 1.2 | 1.9 | 2.7 | 0.0% | 2.1 | 3.1 | 4.2 | 0.0% |
| Yamatokoriyama | 1.2 | 2.0 | 3.0 | 0.0% | 1.9 | 3.0 | 4.2 | 0.0% |
| Tenri | 1.4 | 2.4 | 3.5 | 0.0% | 2.1 | 3.1 | 4.3 | 0.0% |
| Kashihara | 1.1 | 1.7 | 2.5 | 0.0% | 1.9 | 2.8 | 3.9 | 0.0% |
| Sakurai | 1.2 | 2.1 | 3.0 | 0.0% | 1.9 | 3.1 | 4.6 | 0.0% |
| Gojo | 1.4 | 2.5 | 4.3 | 0.0% | 2.3 | 4.0 | 6.3 | 0.0% |
| Gose | 1.3 | 2.4 | 3.4 | 0.0% | 2.6 | 4.3 | 6.8 | 0.0% |
| Ikoma | 1.4 | 2.2 | 3.1 | 0.0% | 2.1 | 3.0 | 4.2 | 0.0% |
| Kashiba | 1.3 | 2.1 | 2.8 | 0.0% | 2.0 | 2.9 | 3.9 | 0.0% |
| Katsuragi | 1.3 | 2.1 | 2.9 | 0.0% | 2.0 | 2.9 | 3.9 | 0.0% |
| Uda | 1.4 | 2.4 | 4.6 | 0.0% | 2.6 | 4.2 | 7.5 | 0.0% |
| Yamazoe | 1.7 | 3.4 | 6.8 | 0.0% | 2.7 | 4.7 | 8.4 | 0.0% |
| Heguri | 1.9 | 2.8 | 3.8 | 0.0% | 4.1 | 5.1 | 7.0 | 0.0% |
| Sango | 1.9 | 2.7 | 3.5 | 0.0% | 2.5 | 3.3 | 4.1 | 0.0% |
| Igaruga | 1.3 | 2.0 | 2.6 | 0.0% | 2.2 | 3.1 | 3.9 | 0.0% |
| Ando | 2.4 | 2.9 | 3.4 | 0.0% | 3.8 | 4.2 | 4.6 | 0.0% |
| Kawanishi | 1.3 | 2.1 | 2.6 | 0.0% | 1.7 | 2.5 | 3.0 | 0.0% |
| Miyake | 1.4 | 2.0 | 2.7 | 0.0% | 1.8 | 2.4 | 3.1 | 0.0% |
| Tawaramoto | 1.4 | 2.3 | 3.1 | 0.0% | 2.3 | 3.4 | 4.4 | 0.0% |
| Soni | 2.2 | 5.4 | 7.3 | 0.0% | 4.1 | 8.2 | 9.8 | 0.0% |
| Mitsue | 1.8 | 3.3 | 5.8 | 0.0% | 4.1 | 6.5 | 9.0 | 0.0% |
| Takatori | 1.2 | 1.9 | 2.8 | 0.0% | 2.7 | 4.1 | 5.4 | 0.0% |
| Asuka | 1.7 | 2.6 | 3.7 | 0.0% | 3.0 | 4.5 | 5.9 | 0.0% |
| Kanmaki | 1.0 | 1.7 | 2.7 | 0.0% | 1.4 | 2.2 | 3.2 | 0.0% |
| Oji | 1.4 | 2.3 | 3.1 | 0.0% | 2.0 | 3.1 | 4.5 | 0.0% |
| Koryo | 1.4 | 2.0 | 2.6 | 0.0% | 2.5 | 3.5 | 4.4 | 0.0% |
| Kawai | 1.0 | 1.6 | 2.1 | 0.0% | 1.5 | 2.1 | 2.6 | 0.0% |
| Yoshino | 1.3 | 2.9 | 4.9 | 0.0% | 4.3 | 8.0 | 10.2 | 0.0% |
| Oyodo | 1.8 | 2.7 | 4.0 | 0.0% | 3.6 | 4.8 | 5.9 | 0.0% |
| Shimoichi | 2.7 | 4.2 | 8.5 | 0.0% | 4.4 | 5.8 | 10.2 | 0.0% |
| Kurotaki | 3.1 | 5.2 | 8.1 | 0.0% | 20.2 | 21.3 | 24.1 | 0.0% |
| Tenkawa | 4.6 | 11.7 | 13.2 | 0.0% | 5.9 | 12.9 | 14.4 | 0.0% |
| Nosegawa | 2.2 | 18.1 | 25.5 | 25.0% | 2.2 | 18.1 | 25.5 | 25.0% |
| Totsukawa | 2.2 | 5.8 | 11.8 | 3.0% | 21.2 | 32.6 | 40.5 | 78.0% |
| Shimokitayama | 2.8 | 6.2 | 10.8 | 0.0% | 5.0 | 8.2 | 10.6 | 0.0% |
| Kamikitayama | 1.3 | 3.5 | 8.0 | 0.0% | 19.7 | 20.7 | 26.1 | 30.0% |
| Kawakami | 6.6 | 11.8 | 14.1 | 1.0% | 16.5 | 30.1 | 32.4 | 56.0% |
| Higashiyoshino | 1.7 | 4.7 | 6.6 | 0.0% | 3.8 | 7.2 | 12.0 | 0.0% |
